# Prevalence of celiac-related antibodies and its impact on metabolic control in Egyptian children with type 1 diabetes mellitus

**DOI:** 10.1186/s12887-024-04575-8

**Published:** 2024-02-05

**Authors:** Khaled A. Abdel Baseer, Abdallah E. Mohammed, Aya Mohamed Abo Elwafa, Hala M. Sakhr

**Affiliations:** 1 Department of Pediatrics, Faculty of Medicine, South Valley University, Qena, Egypt; 2 Department of Clinical Pathology, Faculty of Medicine, South Valley University, Qena, Egypt

**Keywords:** Celiac disease, Metabolic control, Screening, Type 1 diabetes

## Abstract

**Objective:**

The simultaneous presence of celiac disease and type 1 diabetes (T1DM) is coupled with more hazards of comorbidities and complications. This current study aimed to screen for celiac disease in Egyptian children with type 1 diabetes and evaluate its impact on glycemic control.

**Methods:**

A cross-sectional study was verified with 200 Egyptian children diagnosed with T1DM and having a diabetic duration of less than five years. Testing for anti-tissue transglutaminase IgA (tTG-IgA), anti-tissue transglutaminase IgG (tTG-IgG), anti-Endomysial IgA (EMA), and Hb A1c levels were done.

**Results:**

The serological screening revealed that 11 cases (5.5%) tested positive; 8 children with T1DM (4.0%) showed tTG-IgA antibodies ≥ 10 times the upper limit of normal (ULN) with at least one symptom; and 3 cases (1.5%) had levels between 20 and 50 IU/ml (considering a cut-off point of 10 U/ML for positive results). Intestinal biopsy was performed for these three cases, with one case detected to have subtotal villous atrophy, resulting in an overall prevalence of celiac disease in T1DM as 4.5%. Children with positive screening exhibited a higher insulin dose, a higher HbA1c, an increased frequency of hypoglycemic attacks, and recurrent DKA compared to negative cases. A negative correlation was detected between tTG-IgA antibodies with height Z score and hemoglobin level, while a positive correlation was found between tTG-IgA antibodies and HbA1c level.

**Conclusion:**

Undiagnosed celiac disease in children with T1DM negatively impacted metabolic control and affected their general health.

## Introduction

Type 1 diabetes mellitus (T1DM) is among the most recognizable chronic autoimmune diseases present at a young age, involving numerous genetic and environmental risk factors in its pathological process [1]. Children with T1DM face a higher risk of developing celiac disease (CD) compared to the general population [2].

Celiac disease is a hereditary autoimmune condition attributed to the presence of gluten and accompanying prolamines in genetically susceptible individuals. It is accompanied by a variable combination of gluten-dependent clinical presentations, enteropathy, specific antibodies, HLA-DQ2, and HLA-DQ8 haplotypes [3]. Celiac disease and diabetes have multiple involved genetic bases, with over thirty genetic loci implicated in their pathogenesis. The contribution of human leukocyte antigens (HLA) class II genes to hereditary susceptibility has been proven [4]. Celiac disease occurs more frequently in diabetes in comparison to the general populace, with a prevalence of five to seven times higher [5], nearly 5% in diabetes versus nearly 1% in the general populace [6]. The presence of concomitant auto-immune diseases in children with T1DM can complicate diabetes management [7], leading to unsatisfactory metabolic control and a higher risk of having hypoglycemia in comparison to children without concomitant CD [8].

Children with T1DM often exhibit a subclinical or non-classical presentation when associated with celiac disease [9]. The American College of Gastroenterology (ACG) guidelines recommend using tTG IgA as the first-line diagnostic test for children over two years [10]. The American Diabetes Association advises initiating screening soon after the diabetic diagnosis and repeating it after 2–5 years if the previous screening was negative. Symptomatic children with T1DM should be rescreened, even if the previous test was negative [11].

Celiac disease can be considered in children using the no-biopsy approach with a proper high TGA-IgA value (≥ ten times ULN), positive EMA-IgA in a second serum sample, and with at least 1 symptom [12, 13]. In a study by Bybrant et al. [14] conducted with 2035 children and adolescents with T1DM found that TGA-IgA levels ≥ tenfold ULN are associated with celiac disease diagnosis in those children and adolescents. Therefore, they recommended the no-biopsy approach to be applied when indicated as a not intrusive method in T1DM [14].

This current study aimed to screen for celiac disease in Egyptian children with type 1 diabetes to evaluate its impact on glycemic control and children’s growth, with the goal of improving their quality of life.

## Materials and methods

### Study design

A hospital-based prospective cross-sectional study was performed with 200 Egyptian children and adolescents aged from 5 to 18 years old diagnosed with T1DM [15] with a diabetic duration of less than five years. The study participants were recruited from the pediatric endocrinology clinic and pediatric department of Qena University Hospital, South Valley University, Qena, Egypt from January 2021 to June 2022. Cases were collected following the guidelines established in the Declaration of Helsinki. Approval from the institutional Ethics Committee was obtained with ethical approval code SVUMEDPED02542211, and ethical written consent was also obtained from the parents or caregivers of all children involved.

### Clinical evaluation of the participants

The medical history of the participants was taken in detail, including age, time of the first insulin injection (to determine the age of diabetic onset) [16], family history of type 1 diabetes, injected insulin doses and their type, history of recurrent diabetic ketoacidosis (with more than one hospitalization for DKA during the study period, termed recurrent DKA) [17], history of recurrent or unexplained hypoglycemia, family history of celiac disease, history of fatigue, weight loss, musculoskeletal pain, recurrent oral ulceration, hair loss, and nutritional history. Additionally, participants were assessed for gastrointestinal manifestations such as chronic abdominal pain, distension, vomiting, chronic diarrhea, or constipation.

Accurate anthropometric measurements of weight (kg) and height (cm) were taken. Body mass index (BMI) was calculated, and all anthropometric data were recorded precisely and compared with the Egyptian growth curves [18]. The individuals’ measurements were interpreted as standard deviation scores (Z scores), with an expression of the values below or above the reference means or median value [19]. Children were classified as having short stature if their height was ≥ two standard deviations below the mean for children of the same sex and chronologic age [20]. A meticulous evaluation and examination were performed for all participants.

### Laboratory investigations

Before measuring tTG-IgA levels, it was confirmed that children with T1DM had normal IgA levels and consumed a diet encompassing gluten as part of their usual dietary habits. For cases with newly onset diabetes, HBA1c evaluation was considered after at least 3 months of starting insulin therapy.

Blood Sampling: 8 milliliters of venipuncture were gathered using a completely aseptic technique and separated into three test tubes: two tubes containing Ethylenediaminetetraacetic acid (EDTA) for performing complete blood count and HBA1c. Children with T1DM aged 5–12 years and those aged 12–15 years were considered to have anemia with a hemoglobin level < 11.5 and < 12 g/dL, respectively [21]. Children with T1DM were deemed to have good glycemic control when their HbA1c < 7.5% (58 mmol/mol) [22].

The third tube, containing no anti-coagulant (4 ml), was used for the other biochemical testing. The blood in the plain tube was allowed to clot for 30 min in a water bath at 37^o^c. The collected tube was then centrifuged at 3000 xg for 10 min at normal ambient temperature, and the aliquots in Eppendorf were preserved at ^_^ 20 ^o^C until the time of the test assay.

The samples were analyzed for anti-tTG-IgA (Quanta lite R h-tTG IgA), anti-tTG-IgG (Quanta lite R h-tTG Ig G) by enzyme-linked immunosorbent assay, with a cut-off point 10 U/ML for positive results [23]. Additionally, EMA (INOVA Diagnostics Inc., San Diego, California, USA) was analyzed by the indirect immunofluorescence assay (IFA) using a monkey esophagus with a laboratory reference range negative/positive.

### Intestinal biopsy

Cases with persistently elevated Anti-tTG-IgA and less than tenfold ULN had undergone an intestinal biopsy, where 4–6 duodenal biopsies were taken. The specimens were sent to the hospital pathology department for evaluation of the extent of mucosal damage, which was ranked as partial, subtotal, or total villous atrophy.

## Statistical analyses

Statistical analysis was performed with Statistical Package for the Social Sciences (SPSS) software version 26.0 (IBM Corp., Armonk, NY, USA). Data normality was assessed using Kolmogorov-Smirnov and Shapiro-Wilk tests. Normally distributed continuous variables were expressed as mean ± standard deviation (SD), while non-normally distributed continuous variables were expressed as median (Interquartile range). Differences between the two groups were detected using the Independent-Samples T test for normally distributed continuous variables and the Mann-Whitney test was used for non-normally distributed continuous variables. Categorical variables were described by number and percentage, and differences between the two groups were evaluated using the Chi-square test. Spearman’s rank correlation was used to analyze the correlation between non-normally distributed continuous data. A p-value less than 0.05 was considered statistically significant.

## Results

### Baseline characteristics of the study group

This study included 200 Egyptian children with T1DM, with a mean age of 9.39 ± 2.72 years; females represented a significant percentage of cases (54%). The median diabetic duration was 3.0 years with an interquartile range *(IQR)* of (2–4), and the mean age of diabetic onset was 7.78 ± 2.7 years. A family history of T1DM was detected in only 34 cases (17%). Recurrent DKA was observed in 30 cases (15%), while hypoglycemic attacks were observed only in 19 cases (9.5%). All patients were on long and short-acting insulin therapy with a mean insulin dose of 0.97 ± 0.16 U/kg, as illustrated in Table 1.

**Table 1:** Baseline characteristics of the study cases

Variable	Patients (N = 200)	Patients’ subgroups		
		Patients with positive tests (n = 11)	Patients with negative tests (n = 189)	p-value
Age/ year (Mean ± SD)	9.39 ± 2.72	8.41 ± 2.67	9.44 ± 2.72	0.222
Sex (N. %):				
Male Female	92(46%) 108(54%)	3(27.27%) 8(72.73%)	89(47.09%) 100(52.91%)	0.200
Duration of type 1 diabetes/year Median (IQ range)	3.0 (2–4)	2.0 (1–4)	3.0 (2–4)	0.293
Age of diabetic onset/ year (Mean ± SD)	7.78 ± 2.7	5.73 ± 2.66	7.9 ± 2.66	0.009**
Age of diabetic onset (N. %)				
≤ 5 years >5 years	23(11.5%) 177(88.5%)	4(36.4%) 7(63.6%)	19(10.1%) 170(89.9%)	0.029*
Family history of type 1 diabetes				0.473
(N. %) Yes No	34(17%) 166 (83%)	1(9.09%) 10 (90.9%)	33(17.46%) 156 (82.54%)	
Hypoglycemic attacks (N. %)				
Yes No	19(9.5%) 181 (90.5%)	5(45.45%) 6 (54.5%)	14(7.41%) 175 (92.6%)	< 0.001*
Recurrent DKA (N. %)				
Yes No	30(15%) 170 (85%)	5(45.45%) 6 (54.5%)	25(13.23%) 164 (86.8%)	0.004*
Insulin dose U/kg/day (Mean ± SD)	0.97 ± 0.16	1.15 ± 0.24	0.96 ± 0.15	< 0.001*
Weight Z score (Mean ± SD)	-1.29 ± 0.57	-1.14 ± 1.36	-1.3 ± 0.49	0.354
Height Z score (Mean ± SD)	-1.23 ± 0.53	-1.59 ± 0.55	-1.21 ± 0.52	0.018*
BMI Z score (Mean ± SD)	-0.81 ± 0.68	-0.89 ± 0.48	-0.81 ± 0.69	0.681

-P-value between patients with positive tests and patients with negative tests. - *Statistically significant predictor (p < 0.05). -DKA: Diabetic ketoacidosis – BMI: Body mass index

Children with T1DM with positive celiac screening had a mean age of 8.41 ± 2.67 years with 8 cases (72.73%) being females. The age of diabetic onset in children with positive serological tests was significantly lower than in children with negative serological results, with 4 cases (36.4%) having diabetic onset before the age of 5 years old. Additionally, children with positive tests had a significantly higher insulin dose, more hypoglycemia attacks, and recurrent DKA compared to negative cases. Children with T1DM with positive serologic screening were substantially shorter, with a lower height Z score than those with negative screening (see Table 1).

The clinical presentation of cases with positive screening was variable, with abdominal distension and chronic abdominal pain being the most presented manifestations (63.64%). This was followed by chronic fatigue (54.55%), bone pain and anemia (45.45% for both), and lastly short stature, recurrent oral ulcers, and hair loss (Table 2).

**Table 2:** Clinical picture of cases with positive tTG-IgA antibodies

Variable (n. %)	N = 11
Abdominal distension	7(63.64%)
Chronic abdominal pain	7(63.64%)
Fatigue	6(54.55%)
Bone pain	5(45.45%)
Anemia	5(45.45%)
Short stature	2(18.18%)
Hair loss	2(18.18%)
Recurrent oral ulcers	2(18.18%)

### Laboratory data of the included children with T1DM

Laboratory evaluation revealed that good glycemic control was presented in 68 cases (34%) with a mean Hb A1c level of 9.29 ± 1.64. Children with positive screening had significantly higher mean Hb A1c levels (10.74 ± 2.01) than those with negative results (9.21 ± 1.58). Serological screening with positive tTG-IgA, tTG-IgG, and positive Anti-Endomysial IgA antibodies was detected in 11 cases; 8 cases had tTG-IgA antibodies ≥ 10 times ULN with at least one symptom, and 3 cases between 20 and 50 u/ml. children with positive tests had significantly lower hemoglobin levels in comparison to those with negative celiac screening. Additionally, a significant difference between levels of tTG-IgA and tTG-IgG antibodies in the two groups was detected (Table 3).

**Table 3:** Laboratory data of the included children with T1DM

Variable	Patients (N = 200)	Patients’ subgroups		
		Patients with positive tests (n = 11)	Patients with negative tests (n = 189)	p-value
Hemoglobin level (Mean ± SD)	12.65 ± 1.12	11.69 ± 1.42	12.71 ± 1.07	0.003*
Cases with anemia (N. %)	21 (10.5%)	5 (45.5%)	16 (8.5%)	0.0001*
HCT (Mean ± SD)	39.11 ± 3.25	37.46 ± 4.7	39.2 ± 3.14	0.085
MCH (Mean ± SD)	33.4 ± 4.57	30.52 ± 4.06	33.56 ± 4.55	0.031*
MCV (Mean ± SD)	78.62 ± 3.9	77.66 ± 3.17	78.68 ± 3.94	0.404
WBCs (Mean ± SD)	6.27 ± 1.55	6.24 ± 1.65	6.27 ± 1.55	0.942
Platelets (Mean ± SD)	297.34 ± 81.76	316.82 ± 125.47	296.21 ± 78.84	0.418
RBG (Mean ± SD)	242.08 ± 74.18	282.91 ± 124.75	239.7 ± 69.95	0.060
Hb A1c (Mean ± SD)	9.29 ± 1.64	10.74 ± 2.01	9.21 ± 1.58	0.002*
Glycemic control (N. %)				
Good control (HbA1c < 7.5%) Poor control (HbA1c ≥ 7.5%)	68(34%) 132(66%)	2(18.18%) 9(81.82%)	66 (34.92%) 123(65.08%)	0.255
tTG-IgA antibodies IU/ml				
Median (IQ range)	3.0 (1.85–4.3)	230.6(48.7–260.2)	2.8(1.7–4)	< 0.001*
tTG-IgG antibodies IU/ml				
Median (IQ range)	4(2.9–5.7)	38(15–59.7)	3.8(2.65–5.3)	< 0.001*

-P-value between patients with positive tests and patients with negative tests. *Statistically significant predictor (p < 0.05). - HCT: Hematocrit - MCH: Mean corpuscular Hemoglobin- MCV: Mean corpuscular volume – WBCs: White blood cells- RBG: Random blood glucose- Hb A1c: Hemoglobin A1C- tTG-IgA antibodies: anti-Tissue transglutaminase IgA antibody - tTG-IgG antibodies: anti-tissue transglutaminase IgG Antibody

### Correlations of tTG-IgA antibodies with clinical and laboratory data in children with T1DM

**Fig. 1:**
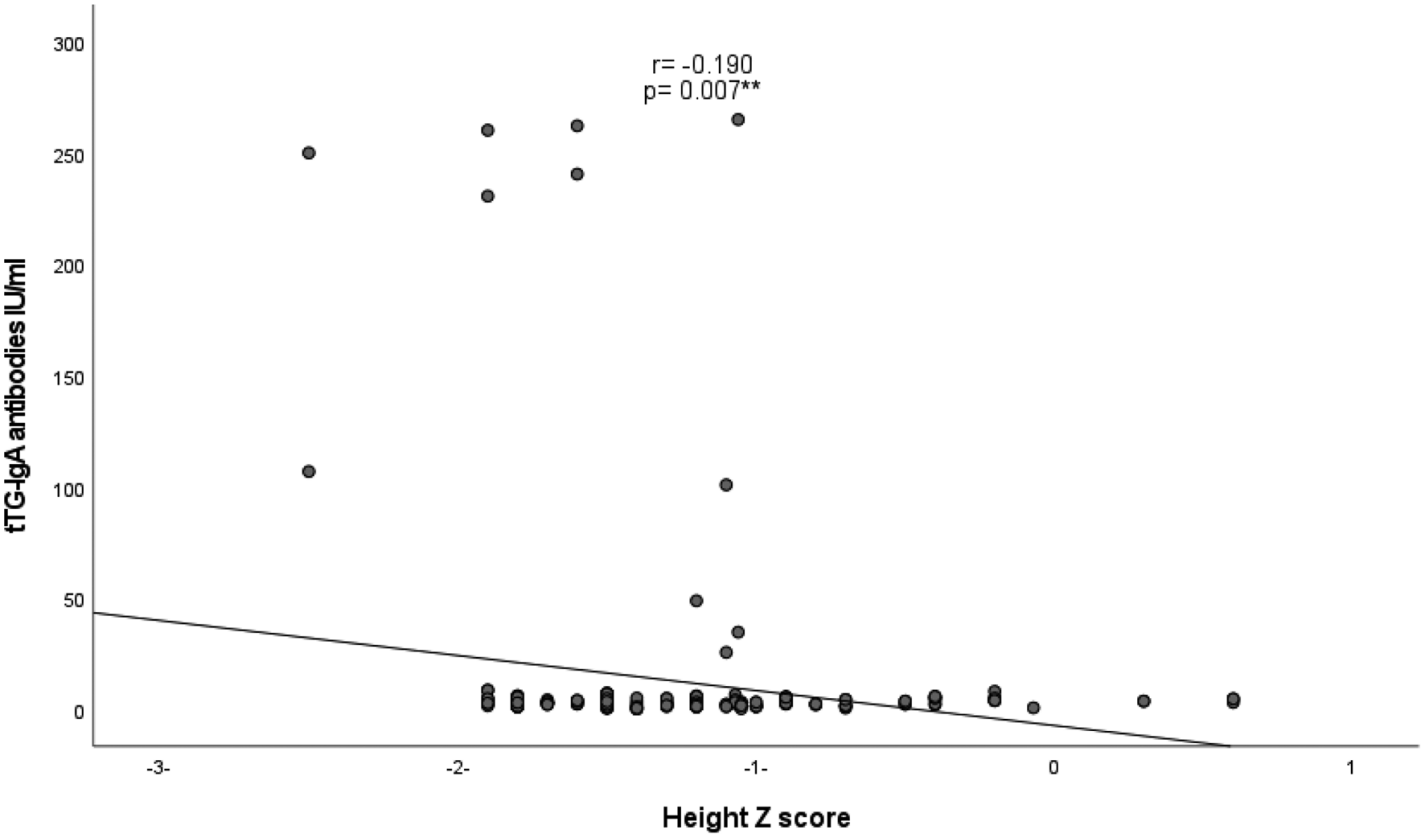
Correlation between tTG- IgA antibody levels and height Z score

**Fig. 2:**
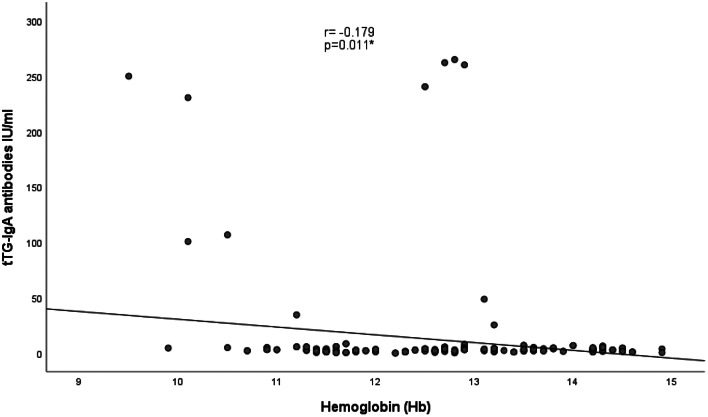
Correlation between tTG- IgA antibody levels and hemoglobin levels

**Fig. 3:**
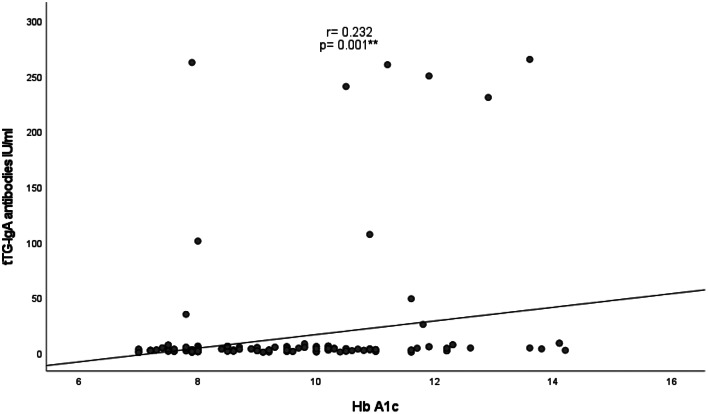
Correlation between tTG- IgA antibody levels and Hb A1c levels

As presented in Figs. 1 and 2, negative correlations were detected between tTG-IgA antibodies and height Z score (r = − 0.190, and p = 0.007) and hemoglobin level (r = − 0.179, p = 0.011). A positive correlation was also identified between tTG-IgA antibodies and HbA1c (r = 0.232, p = 0.001), as illustrated in Fig. 3.

### Intestinal biopsy

**Fig. 4:**
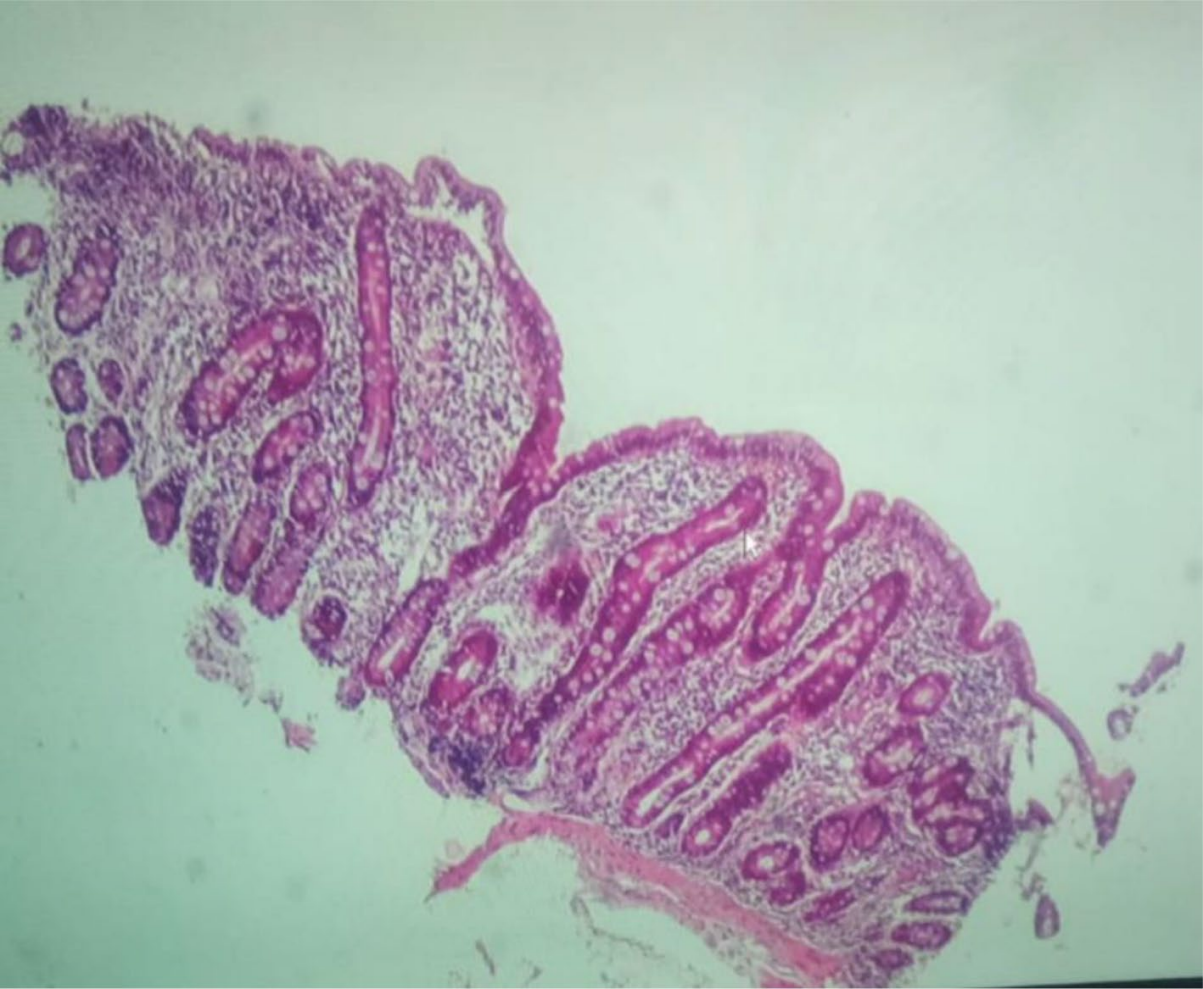
Subtotal villous atrophy in intestinal biopsy

Duodenal biopsies were performed for the three cases with positive EMA test and the tTG-IgA antibody levels between 20 and 50 IU/ml. One case was detected to have subtotal villous atrophy (Fig. 4), while the others showed nonspecific duodenitis for follow-up.

## Discussion

The current study screened 200 children with T1DM for the concomitant presence of celiac antibodies. Children with positive screening were found to be shorter and had significantly higher insulin doses, more hypoglycemia attacks, and recurrent DKA compared to negative cases. Celiac disease is a serious autoimmune gluten-sensitive chronic enteropathy that exists more frequently in T1DM than in the general population [24]. Antibody testing against gliadin and deamidated gliadin peptide for the diagnosis of celiac disease has been substituted by tTG-IgA antibodies and EMA testing, which both have higher specificity and sensitivity [25].

The current study found that the overall prevalence of celiac disease in T1DM was 4.5%. This is consistent with the results of a large cross-sectional study conducted by Taczanowska et al. [26], which reported a 4.5% prevalence of celiac disease in children. Another two large meta-analyses conducted by Gheshlagh et al. [27] and Albatineh et al. [28] revealed a prevalence of CD in children with T1DM was 5% and 5.08% respectively.

Karsas et al. [29] detected a positive antibody screening for CD in 10.2% of T1DM. In a large retrospective study, Wędrychowicz et al. [30] concluded that celiac disease was diagnosed in 8.3% of cases and most cases were asymptomatic.

The European Society for Pediatric Gastroenterology, Hepatology, and Nutrition (ESPGHAN) proposed that intestinal biopsies could be avoided for symptomatic children older than 2 years old with the tTG-IgA antibodies ≥ tenfold ULN [3]. Mubarak et al. [31] and Webb et al. [32] also found that screened children with high anti-TtG levels were confirmed to have celiac disease through intestinal biopsy. Wolf et al. [33] confirmed and supported the precise celiac diagnosis in screened children with T1DM without performing the biopsies, and Paul et al. [34] concluded that children with T1DM should be incorporated into the ESPGHAN guidelines protocols for Celiac diagnosis.

Bybrant et al. [14] and, Taneja et al. [35] revealed that tTGA ≥ 10 times ULN is correlated to the degree of mucosal damage with a Marsh grade > stage II.

In the present study, celiac disease was observed more frequently among females. Multiple studies supported these findings [26, 29, 30], while others reported the absence of sex differences in their studied population [36, 37] or demonstrated a male predominance [38–40].

Our study documented a lower age of diabetic onset among children with positive serological tests compared to those with negative tests (5.73 ± 2.66 years vs. 7.9 ± 2.66 years). These results were consistent with previous researchers [26, 30].

In the current study 5 patients (45.5%) who tested positive presented with anemia, compared to 16 children (8.5%) with negative tests. The positive cases exhibited significantly lower hemoglobin levels than those with negative celiac screening, and a negative correlation was detected between tTG-IgA antibodies and hemoglobin levels. In agreement with these results, Joshi et al. [41] observed that 54.5% of diabetic cases with CD had anemia as compared to 20% of those without CD with a statistically significant difference. Pulikkal et al. [42] also found a significant negative correlation between IgA tTG positivity with hemoglobin levels. Iron deficiency anemia is a frequent finding in celiac patients [43], possibly attributed to defective duodenal mucosal iron absorption and occult gastrointestinal bleeding [44].

In the current study, seropositive children had higher insulin doses, more frequent hypoglycemic attacks, recurrent DKA, and a higher HbA1c compared to negative cases. The study demonstrates a positive correlation between tTG-IgA antibodies, insulin dose, and HbA1c.

Various studies have yielded controversial results regarding the effect of celiac disease on metabolic control. Some studies [30, 37, 45] couldn’t find a significant difference in the metabolic control, while in concordance with our results, Albatayneh et al. [23] noted a higher baseline glycated hemoglobin (HbA1c) in seropositive patients. Taczanowska et al. [26] found that children with T1DM and celiac disease used glucose monitoring equipment and insulin pumps more frequently in contrast to patients without CD. Additionally, Khoury et al. [7] documented the presence of unexplained severe hypoglycemia in young children with T1DM with celiac disease.

Several authors have investigated the effect of the gluten-free diet (GFD) on children with T1DM and celiac disease. Hansen et al. [46] revealed a clinical improvement in these children, and in a randomized control trial, Kaur et al. [47] documented a tendency for a decrease in hypoglycemic attacks and improvement in glycemic control in patients receiving GFD. Söderström et al. [48] also found an association between GFD compliance, glycemic control, and the frequency of DKA attacks, highlighting that active celiac disease could negatively affect glycemic control [49].

## Limitation of the study

Intestinal biopsies were limited to cases with persistently elevated tTG-IgA and less than 10 times ULN as it is an invasive, challenging, costly procedure with the added risks of general anesthesia and prolonged fasting period for diabetic patients.

## Conclusion

The overall prevalence of celiac disease in children with T1DM was 4.5%. Seropositive children are shorter and have lower hemoglobin levels compared to seronegative cases. Celiac disease negatively impacts metabolic control and influences children’s growth. Regular screening for concomitant celiac disease with T1DM is recommended for early diagnosis and adequate treatment, with close follow-up of patients’ growth.

## Data Availability

The datasets used and/or analyzed during the current study are available from the corresponding author upon reasonable request, after obtaining the permission of our institute.

## Abbreviations

ACG: American College of Gastroenterology
CD: Celiac disease
EMA-IgA: Endomysial antibodies
ESPGHAN: European Society for Pediatric Gastroenterology, Hepatology, and Nutrition
GFD: Gluten-free diet
HLA: Human leukocyte antigens
IFA: Indirect immunofluorescence assay
SPSS: Statistical Package for Social Science
tTG IgA: Anti-tissue transglutaminase immunoglobulin A
T1DM: Type 1 diabetes mellitus
ULN: upper limit of normal
